# Exploring Protective Factors in Wellbeing: How Sensory Processing Sensitivity and Attention Awareness Interact With Resilience

**DOI:** 10.3389/fpsyg.2021.751679

**Published:** 2021-11-16

**Authors:** Bożena Gulla, Krystyna Golonka

**Affiliations:** Institute of Applied Psychology, Faculty of Management and Social Communication, Jagiellonian University, Kraków, Poland

**Keywords:** sensory processing sensitivity, highly sensitive person, highly sensitive person scale, attention awareness, mindfulness, resilience

## Abstract

The aim of the study is to analyze the relationship between sensory processing sensitivity (SPS), attention awareness, and resilience and to determine whether attention awareness may moderate the relation between sensitivity and resilience. The sample consisted of 273 adults (239 women; *M*_age_=24.12, *SD*=6.59years). The *highly sensitive person scale*, the *resiliency assessment scale*, and the *mindful attention awareness scale* were used in the study. The results indicate significant relationships between SPS and resilience; emotional reactivity is especially associated with lower resilience, whereas sensing the subtle is associated with higher resilience. The regression analyses revealed that SPS is a significant predictor of resilience, but diverse aspects of sensitivity explain resilience differently. Attentional awareness was found to be a significant moderator that strengthens the positive relationship between sensing the subtle and tolerance of negative emotions. The consequences of high sensitivity include high levels of distress, anxiety, and a sense of overload; therefore, searching for protective factors is important to maintain the wellbeing of highly sensitive people. As one of the characteristics of highly sensitive persons, sensing the subtle may be an important resource that allows to them to deal effectively with difficult situations. Training on attention awareness and conscious presence may be an important way to deal with negative emotions and develop personal competences. The results indicate that these strategies may be of high significance for improving wellbeing and protecting highly sensitive persons against various stress factors.

## Introduction

The aim of this study is to explore protective factors in wellbeing by analyzing how sensory processing sensitivity (SPS) and attention awareness interact with resilience. Some individual characteristics (e.g., neuroticism) help to predict how a person reacts in various stressful situations, including work-related stress (Leiter and Maslach, 2004). In the context of organizational psychology, analyzing the relationships between SPS and resilience, as well as the possible moderating effects of cognitive resources, may help to define strategies that regulate stress. This analysis may be of high significance in protecting wellbeing among highly sensitive persons.

Elaine Aron’s (Aron and Aron, 1997; Aron, 2017) high-sensitivity construct concerns people with specific nervous system properties who are characterized by high sensitivity of sensory processing that results in some positive consequences but can also lead to a feeling of being overwhelmed and exhausted. SPS is a stable trait characterized by greater awareness, responsiveness, and depth of information processing. It is the way in which sensory information is acquired, selected, and processed by the central nervous system. It is linked with both greater sensitivity to exposure to negative stimuli and better use of positive aspects of situations and interactions (Aron et al., 2012). High sensitivity can be an advantage in ensuring careful, safe, mindful, and effective functioning. Highly sensitive people are characterized by the DOES acronym (Aron, 2017): D – depth of processing, O – overstimulation, E – emotional reactivity, and S – sensing the subtle. Highly sensitive people are one of three groups into which the population can be divided (Lionetti et al., 2018; Pluess et al., 2018): The floral metaphor (Lionetti et al., 2018) distinguishes *Orchids* (high level of sensory processing), *Tulips* (moderately sensitive), and *Dandelions* (lowest level of sensitivity).

Sensory processing sensitivity “is proposed to be a genetically determined trait involving a deeper (…) cognitive processing of stimuli that is driven by higher emotional reactivity” (Aron et al., 2012, p. 262). The authors refer to Craik and Lockhart’s (1972) concept of processing levels, in which the main thesis is the assumption that each piece information is processed by the same brain structures but at different depths of processing, which means different processing intensities. The first level of processing relates to the physical properties of stimuli: It enables their registration and the detection of differences between them. The second, deeper level of processing includes interpretation of the meaning of stimuli and allows for the semantic categorization of objects that are in the same category. The deepest level of processing is related to the inclusion of associations, memories, and elements of knowledge in information processing; this not only allows for more complete interpretation of incoming data but also enriches knowledge by expanding or creating new cognitive structures. At deeper levels, processing takes longer, but the resistance to disturbance is greater and the results of this process are more durable. Information can be processed in the “primary circuit” (from the first to the deepest level of processing), but it can also be processed in the “secondary circuit” (data can be processed at any level). Not all information reaches the deepest level of processing. Information that is in the “secondary circuit” at any level of processing may remain there or leave it, but the direction of transfer tends toward deeper levels (Nęcka et al., 2020). High sensitivity causes increased susceptibility to external and internal stimuli, manifested in the form of deep processing of sensory information. It also entails susceptibility to being overwhelmed in conditions of overstimulation and may be associated with high levels of stress, ease of exhaustion, depression, anxiety, sleep disorders, and psychophysical disorders. At the same time, however, sensitive people may display good intuition and a high level of integrity and creativity (Aron, 2017).

The sensitivity of sensory processing is described as the interface between individual neurological functioning and the environment; it places individuals on a continuum of varying intensity responses to environmental stimuli (Dean et al., 2018). SPS is conceptualized as a meta-trait. Key to this conceptualization is the fact that high sensitivity is important not only for understanding maladaptation, behavioral disturbances, and psychophysical symptoms as the effects of the influence of an unfavorable environment, but also for optimal development and even thriving in a positive environment (Aron, 2017).

High sensitivity is associated with the dominance of the control pause system and the tendency to stop before taking action (Aron, 2017). This tendency may be referred to as Gray’s concept of the behavioral inhibition system (BIS) and anxiety (Gray, 1982). Behavioral systems are disposed to approach stimuli that are beneficial from the point of view of life needs (Behavioral Activation System – BAS), but they avoid or move away from harmful or threatening stimuli (BIS). The observed behavior is the result of the proportion of BIS and BAS activity. These approach-avoidance tendencies determine the adaptive and evolutionary value of behavioral systems. BIS is sensitive to aversive stimuli (e.g., signals of punishment and lack of reward), innate anxiety stimuli, and new, surprising, or intense stimuli. In the revised theory of Gray’s reinforcement sensitivity theory (Gray and McNaughton, 2000; McNaughton and Corr, 2008), BIS is responsible for resolving goal conflicts; it inhibits conflicting behavioral tendencies and enables the inclusion of situation analysis processes. The probability of the dominance of the BIS in highly sensitive persons may explain why high sensitivity is linked to anxiety and depression (Liss et al., 2005).

Environmental challenges vary greatly: In some situations, quick, direct, or even confrontational action is more beneficial; in others, careful reflection and calm planning will bring better results. As a result of the control pause, highly sensitive people are often more cautious: They compare the current situation with past experiences, analyze all its nuances, and react in a more restrained way. They are characterized by reflection, a high level of empathy, sensitivity to injustice, creativity, perceiving beauty in nature and art, and intuitiveness (Aron, 2017). However, highly sensitive people are perceived by others as shy, unambitious, and withdrawn, which may cause difficulties in building positive self-esteem. Excessive stimuli, especially those of a social nature, make them feel overwhelmed and have a tendency to avoid similar situations. If they have developed in a favorable family environment, it is more likely that they will develop their full potential and will be able to organize contexts appropriate for their needs and capabilities in adulthood (e.g., at work). But if the family environment was not favorable and did not help them build self-acceptance, they may consider sensitivity to be a weakness; they might strive to overcome it but not be able to find the right conditions for themselves, thereby constantly experience overwhelming irritation, tension, and dissatisfaction (Aron, 2017).

Research findings indicate that high sensitivity is not the opposite of resilience (Belsky and Pluess, 2009; Pluess and Belsky, 2013). Highly sensitive persons can cope with difficulties by using their strengths, such as perceiving information with greater precision, subtle differentiation, deeper processing, and noticing nuances. Their reflective attitude allows them to combine their knowledge with data from previous experiences; they observe situations from a broader perspective (time and situational); they carefully analyze and notice many aspects of a situation; and they accept what is inevitable and build action plans for changes. If highly sensitive persons are aware of their disposition, they are able to regulate their emotions effectively using strategies, such as cognitive reformulation, taking another perspective, and humor. They can even adapt to extremely difficult situations by referring to transcendent values (Belsky and Pluess, 2009; Pluess and Belsky, 2013).

The concept of resilience (Luthar and Cicchetti, 2000) relates to positive adaptation and successful coping despite adversities, such as chronic stress. Rees et al. (2015) emphasize that psychological resilience is a predictor of coping strategies in the workplace and is the most important determinant that influences the risk of burnout, compassion fatigue, anxiety, and depression among employees: the higher the resilience, the lower the risk of negative consequences. Mental resilience determines the process of flexible adaptation to the constantly changing requirements of life, in which resilient people show positive adaptation both in traumatic situations and in everyday struggles with adversities. Tronick and DiCorcia (2015) developed the hypothesis of resilience to everyday stress, which assumes that resilience can be treated as a regulation process in coping with cumulative stressors in everyday life. Luthar and Cicchetti (2000) treat resilience as a dynamic disposition that can be continuously potentiated. Resilience is a two-dimensional construct that includes (1) exposure to adversity – negative life circumstances associated with the risk of adaptation difficulties; (2) positive adaptation – usually defined in behavioral categories of manifested social competences. Resilience is a broader category than personality traits as it covers cognitive (beliefs and expectations) and affective (dominant emotions) aspects, regulatory, and coping strategies, as well as social competences that facilitate functioning (e.g., Tebes et al., 2004). Development of mental resilience is a dynamic process that is ongoing throughout life in interactions with environmental conditions and in constant adaptation to environmental challenges (e.g., Liu et al., 2017; Neenan, 2017). For resilient functioning, the proportion of risk factors and protective factors (intrapsychic, interpersonal, and social) is important. Mental resilience also means recovering and regaining balance after painful experiences, and getting through difficult situations without serious negative psychological consequences. Various elements are emphasized in the theory of resilience, e.g., self-efficacy (Bandura, 1993), commitment, a sense of control and treating difficult situations as challenges (Kobasa, 1985), and a sense of coherence and hardiness (Antonovsky, 1987; Almedom, 2005).

Cognitive aspects play a significant role both in SPS and in resilience. Aron (2017) emphasizes that the characteristics of highly sensitive persons include deep processing, a tendency to analyze, and sensitivity to environmental stimuli. Mak et al. (2011) indicate positive thoughts about the self, others, and the future as fundamental characteristics of mental resilience. Rees et al. (2015) presented a model in which mindfulness is a significant variable that influences psychological resilience and psychological adjustment at work. In relation to varying degrees of psychological awareness, Rees et al. (2015) indicate the ability to reflect on an ongoing situation and regulate one’s emotional state, which may be a significant predictor of burnout symptoms. Garland et al. (2011) indicate that positive reappraisal is a mediator between stress reduction and mindfulness.

Mindfulness is defined as a state of consciousness in which attention is focused on the “here and now,” in which the reception of external stimuli, bodily sensations, emotions, thoughts, and ideas are registered and observed but are not assessed or judged; they “flow freely” in the subject’s consciousness (Kabat-Zinn, 1990, 2003). In a state of mindfulness, awareness of the inner world and the external environment is deeper, fuller, and more nuanced; more peripheral stimuli are registered. Mindfulness is associated with conscious presence, low reactivity, and the ability to describe, name, and observe (Radoń, 2014); it also combines inner peace, mental toughness, and endurance. Mindfulness training brings cognitive benefits and helps in coping with stress.

There is some evidence that the brain activity of attentive people has a specific pattern. According to Davidson and Begley (2013), open, non-judgmental awareness is related to specific patterns of brain functioning. Mindfulness-based stress reduction strengthens the activation of the left prefrontal cortex; practicing mindfulness enhances prefrontal cortex control over the functioning of the neural pathways responsible for attention; and it mainly does so by strengthening the connections between the prefrontal cortex and other areas of the brain involved in attention and information processing. Due to the neuroplasticity of the brain, it is possible that the changes that result from frequent mindfulness training are not only functional but also structural. The “mindful” brain is linked to an adaptive and functional emotional style, thus mindfulness training may be a significant emotion regulation strategy (Davidson and Begley, 2013). Mindful attention awareness may also be linked with SPS and resilience; it moderates future negative consequences in adverse environmental conditions. This general association should be emphasized among employees and taken into account in prevention and intervention programs that are designed to reduce the possible health consequences of work-related stress. For highly sensitive young people, joining the labor market may be associated with a high level of stress. Analysis of the factors that increase resilience may allow cognitive strategies to be implemented (or at least considered) that facilitate young persons’ job searches and adaptation to new professional circumstances, thus increasing wellbeing in future employment.

Understanding individual temperamental predisposition is important at any stage of employment, but it may be particularly valuable and profitable in the early stage, i.e., during the transition to the labor market. In this period, highly sensitive young adults may especially need emotional support with identifying their own dispositions, making choices, and making decisions considering the specificity of their functioning. The transition from education to professional work may be associated with high stress as it is a new unknown situation of great importance for further professional development and living conditions. Taking jobs for which they are over-qualified may have a negative influence on young adults’ self-esteem and significantly impact their future professional career and aspirations. Negative experiences when job-seeking may cause states of discouragement, anxiety, loss of self-confidence, symptoms of depression, and even suicidal thoughts (Lim et al., 2018). On the other hand, early unemployment may have long-term consequences, including the risk of future unemployment, lower earnings, discouragement, inability to acquire new skills, or unsatisfactory results during subsequent job interviews (Bradley and Nguyen, 2003). Therefore, it seems particularly important to consider the factors that could protect young employees’ wellbeing.

Analysis of the relationships between sensitivity, mindfulness, and resilience may help to determine the regulating strategies that are crucial for facilitating adaptive ways of functioning in a demanding environment. It was assumed that the moderating variable between a constant trait that results from the specificity of the nervous system (SPS) and mental resilience (a disposition that can be developed) is mindfulness, which may be specific to highly sensitive people due to their increased depth of processing, sensing of nuances, and reflectiveness.

## Research Model and Methods

**Figure 1:**
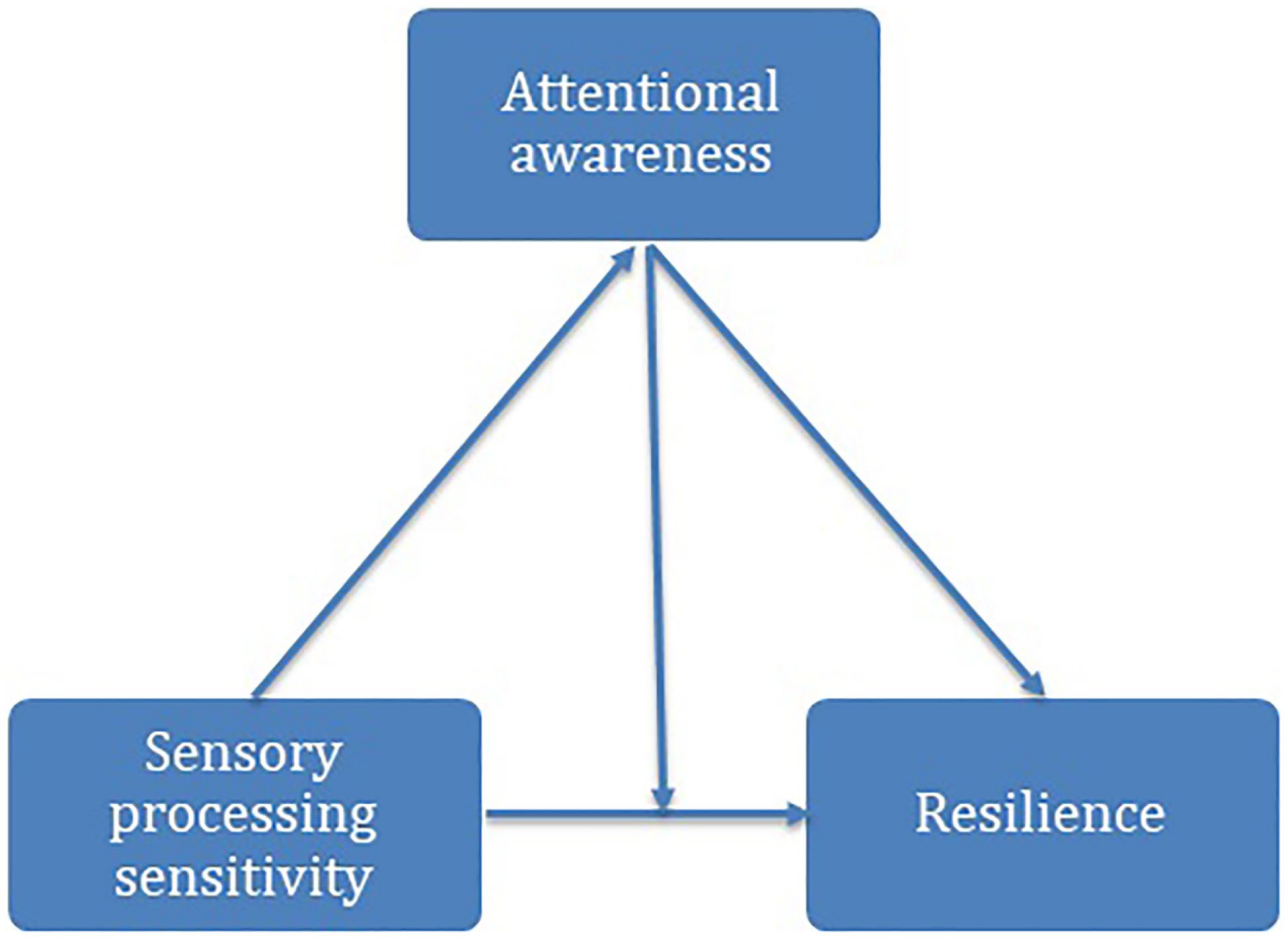
Theoretical model of the relationships between sensory processing sensitivity (SPS), attentional awareness, and resilience.

Regarding the influence of cognitive processes on emotion regulation and coping with stress, in this work, we will analyze the relation between specific aspects of SPS and resilience; we will also test the possible moderating role of mindful attention awareness (Figure 1). The following hypotheses were formulated as:

H1: *The higher the level of emotional reactivity, the lower the level of resilience*.

H2: *The higher the level of overstimulation, the lower the level of resilience*.

H3: *The higher the level of sensing the subtle, the higher the level of resilience*.

H4: *Sensory processing sensitivity and mindful attentional awareness are significant predictors of resilience*.

H5: *Attentional awareness is an important factor that moderates the relationship between sensory processing sensitivity and resilience*.

In the exploratory analysis, we will also test the relationships between attentional awareness and SPS. To test the hypotheses, correlation (H1–H3), regression (H4), and moderation analyses (H5) will be performed.

**Figure 2:**
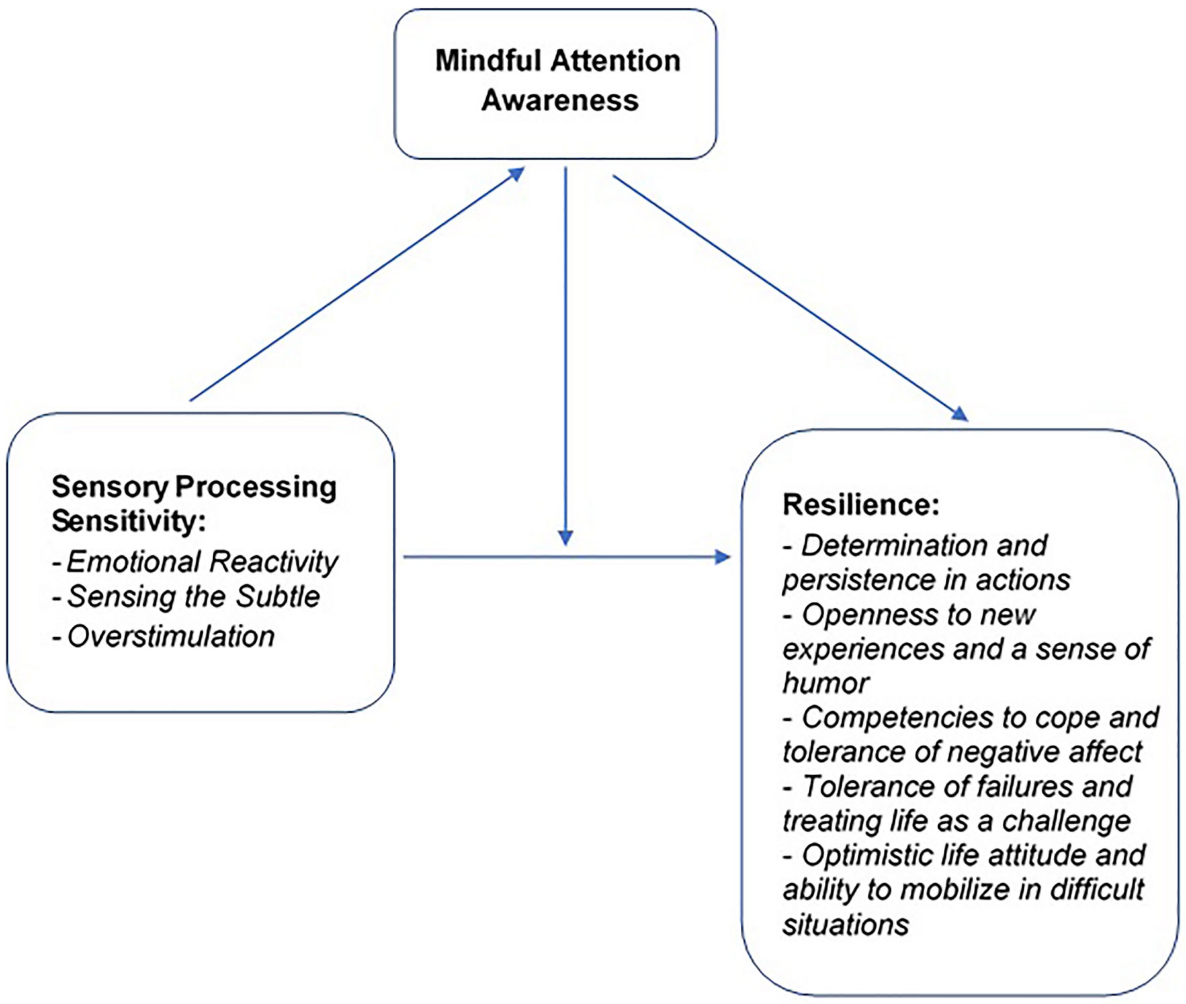
The research model of the tested variables and their relationships.

The research model and the specifications of the tested variables are presented in Figure 2.

### Participants and Procedure

The sample consists of 273 young adults (239 women, 87.5%; 25 men, 9.2%; and 9 other, 3.3%). The study was targeted at young people; therefore, the participants were mainly students (66.3%) and graduates (26.7%). The mean age was *M*_age_=24.12years, *SD*=6.59years. Most participants were single (57.9%) or in an informal relationship (35.5%).

The study was voluntary; the procedure was conducted in accordance with the recommendations of the Helsinki declaration and was accepted by the Research Ethics Committee of the Institute of Applied Psychology, Jagiellonian University in Kraków. The research was conducted online using the university servers. Participants were invited to the study *via* social media and the official Web site of the Institute of Applied Psychology of Jagiellonian University. Thus, the study sample represents a university population prior to joining the workforce: Participants were mainly psychology students, whose characteristics are typical of such a sample, i.e., the sample consists mainly of women.

### Questionnaires

Three self-report instruments were used in the study: the *highly sensitive person scale* (HSPS) to measure the level of SPS; the *mindful attention awareness scale* (MAAS) to measure cognitive characteristics; and the *resiliency assessment scale* (RAS) to measure resilience.

The HSPS by Aron and Aron (1997) consists of 27 items that are answered on a 7-point scale from 1 (*not at all*) to 7 (*extremely*). The Polish version was developed by the authors of this work. Smolewska et al. (2006) indicate that HSPS has a three-factor structure: *Ease of Excitation* (EOE, 12 items), *Aesthetic Sensitivity* (AES, 6 items), and *Low Sensory Threshold* (LST, 7 items). In Smolewska et al.’s study, the three distinguished subscales explained 40.5% of the total variance and demonstrated strong reliability: Cronbach’s alpha of 0.89. The three-factor structure of the scale has been confirmed in many studies (Smolewska et al., 2006; Evers et al., 2008; Grimen and Diseth, 2016; Lionetti et al., 2018). There are also one-factor (Aron and Aron, 1997), two-factor (Evans and Rothbart, 2008; Ershova et al., 2018), and even six-factor (Blach, 2015) solutions. In the presented study, Cronbach’s alpha was 0.88. We demonstrate the three-factor solution, which explains 41% of variance. The factors distinguished in this analysis are similar to those presented by Smolewska et al. (2006). When analyzing the content of the items, we abstracted the following subscales: (1) *Emotional Reactivity* (ER, 12 items); (2) *Sensing the Subtle* (StS, 6 items); and (3) *Overstimulation* (OvSt, 7 items).

Brown and Ryan’s (2003) MAAS consists of 15 items that are answered on a 6-point scale, from 1 (*almost always*) to 6 (*almost never*). The Polish version was developed by Radoń (2014) and has satisfactory reliability: Cronbach’s alpha 0.81–0.85; stability 0.81–0.91. The scale can be used for people who have had no previous experience in practicing mindfulness. Mindful attention awareness is measured by MAAS as a one-factor construct; it is defined as a receptive state of attention which through awareness of current experiences enables open observation of what is happening (Brown and Ryan, 2003; Radoń, 2014). Research by Radoń (2014) indicates that mindfulness is significantly negatively related to level of rumination (*r*=−0.33; *p*<0.01), neuroticism (*r*=−0.26; *p*<0.01), emotional instability (*r*=−0.25; *p*<0.01), lack of personality integration (*r*=−0.24; *p*<0.01), and personality disorders (*r*=−0.21; *p*<0.01); mindfulness is significantly positively related to openness to experience (*r*=0.34; *p*<0.01) and reflexivity (*r*=0.11; *p*<0.01).

Ogińska-Bulik and Juczyński’s (2008) RAS was used to measure resilience to stress. RAS consists of 25 items that are answered on a 4-point scale, from 1 (*definitely not*) to 4 (*definitely yes*). Cronbach’s alpha is 0.89 for the entire scale. The internal stability was measured with a retest after 4weeks and is 0.85. Factor analysis revealed that the scale has a five-factor solution. The scale includes the following subscales: (1) *Determination and persistence in actions*; (2) *Openness to new experiences and a sense of humor*; (3) *Competencies to cope and tolerance of negative affect*; (4) *Tolerance of failures and treating life as a challenge*; and (5) *Optimistic life attitude and ability to mobilize in difficult situations*. The reliability of the five separate subscales ranges from 0.67 to 0.75. Each subscale consists of five items.

### Data Analysis

The analysis was conducted using SPSS version 27 (IBM SPSS Statistics, IBM Corporation, United States) with the Proces_v3.5 module (Hayes, 2018). Correlation and regression analyses were performed to explore the relationships between SPS, mindful attention awareness, and resilience. Then, the moderation analysis was performed to explain the role of mindful attention awareness in the relationships between various aspects of SPS and resilience.

## Results

The Kolmogorov-Smirnov tests showed that most of the tested variables did not meet the assumption of normality; therefore, Spearman’s test was used for bivariate correlation to test hypotheses H1–H3. As the analyses of normal Predicted Probability (P-P) plots revealed that the residuals were normally distributed, and the assumptions of linearity, homoscedasticity, and the absence of multicollinearity (correlation between predictors lower than 0.08; variance inflation factor values below 5.00) were met, multiple linear regression and moderation analyses were performed to test hypotheses H4–H6 (Dawson, 2014).

The three components of the Polish version of HSPS accounted for 41% of the variance (eigenvalues: 7.18, 2.21, 1.69). The abstracted subscales were defined considering the content of the items and the DOES highly sensitive person model (Aron, 2017). SPS is defined by the following subscales: (1) *Emotional Reactivity* (ER); (2) StS; and (3) OvSt.

### Correlation Analysis

The global HSPS score is significantly and negatively correlated with the overall result of the resilience scale and its four subscales: *Openness to new experiences and a sense of humor*; *Competencies to cope and tolerance of negative affect*; *Tolerance of failures and treating life as a challenge*; and *Optimistic life attitude and ability to mobilize in difficult* situations (Table 1).

**Table 1:** Descriptive statistics, Cronbach alpha, and the results of rho Spearman correlation between the scores of the highly sensitive person scale (HSPS), the mindful attention awareness scale (MAAS), and the resiliency assessment scale (RAS).

S.no	Variable	*M*	*SD*	*α*	1	2	3	4	5	6	7	8	9	10	11
1	HSPS	5.12	0.71	0.88	–										
2	ER	5.28	0.80	0.83	0.85^***^	–									
3	StS	5.41	0.80	0.71	0.57^***^	0.30^***^	–								
4	OvSt	4.77	0.79	0.79	0.87^***^	0.60^***^	0.38^***^	–							
5	MAAS	3.97	0.69	0.84	0.06	−0.07	0.18^**^	0.08	–						
6	RAS	2.47	0.55	0.90	−0.17^**^	−0.39^***^	0.22^***^	−0.07	0.18^**^	–					
7	DPA	2.39	0.75	0.79	0.09	−0.13^*^	0.35^***^	0.15^*^	0.21^***^	0.63^***^	–				
8	OH	2.99	0.59	0.59	−0.16^**^	−0.32^***^	0.15^*^	−0.10	0.22^***^	0.78^***^	0.29^***^	–			
9	CNA	2.31	0.71	0.74	−0.23^***^	−0.39^***^	0.14^*^	−0.12^*^	0.05	0.85^***^	0.44^***^	0.61^***^	–		
10	TFC	2.62	0.65	0.68	−0.17^**^	−0.31^***^	0.15^*^	−0.11	0.25^***^	0.79^***^	0.37^***^	0.63^***^	0.60^***^	–	
11	OM	2.05	0.74	0.74	−0.22^**^	−0.39^***^	0.08	−0.10	0.05	0.88^***^	0.44^***^	0.64^***^	0.75^***^	0.66^***^	–

*In HSPS: ER – Emotional Reactivity; StS – Sensing the Subtle; OvSt – Overstimulation. In RAS: DPA – Determination and persistence in actions; OH – Openness to new experiences and a sense of humor; CNA – Competencies to cope and tolerance of negative affect; TFC – Tolerance of failures and treating life as a challenge; and OM – Optimistic life attitude and ability to mobilize in difficult situations. (N=273)*.

* *p<0.05*;

** *p<0.01*;

*** *p<0.001*.

The correlation coefficients obtained for the HSP scale subscales showed that different aspects of sensitivity relate differently to resilience. While *Emotional Reactivity* and *Overstimulation* were negatively related to almost all resilience subscales (except for a weak positive correlation between *Overstimulation* and *Determination and persistence in actions*), the HSPS *Sensing the Subtle* subscale showed completely different relations: significant positive relationships with the general resilience score and the subscales of *Determination and persistence in actions* (the strongest correlation), *Openness to new experiences and a sense of humor*, *Competencies to cope and tolerance of negative affect*, and *Tolerance of failures and treating life as a challenge* (in these relationships, significant but weak correlations were observed).

The analysis of the correlation coefficients gives support for hypothesis 1 (H1: *The higher the level of emotional reactivity, the lower the level of resilience*) and hypothesis 3 (H3: *The higher the level of sensing the subtle, the higher the level of resilience*).

The correlation between *Overstimulation* and the global score of the RAS, as well as the correlation, among the three RAS subscales was not significant; the significant correlation coefficients between *Overstimulation* and other RAS subscales were ambiguous: *Overstimulation* positively correlated with *Determination and persistence in actions*, but it negatively correlated with *Competencies to cope and tolerance of negative affect*; however, both correlations are weak. Thus, hypothesis 2 (H2: *The higher the level of overstimulation, the lower the level of resilience*) is not confirmed.

Moreover, significant positive correlations were observed between the *Sensing the Subtle* subscale and the *Mindful Attention Awareness* subscale. Significant positive relationships were found between attention awareness and the general resilience score as well as the RAS three subscales: *Determination and persistence in actions*, *Openness to new experiences and a sense of humor*, and *Tolerance of failures and treating life as a challenge*.

The obtained results might suggest that high sensitivity is linked with diminished resilience: The overall HSPS score negatively correlates with the overall RAS score. However, a negative relationship was observed only in one HSPS subscale, i.e., *Emotional Reactivity*, and this correlation is stronger than the overall HSPS score. A completely opposite relationship was observed between the HSPS *Sensing the Subtle* subscale and general resilience. This may indicate that if negative emotionality is dominant in one’s “image” of one’s high sensitivity, it actually may reduce the adaptive potential and the ability to cope with difficult situations. However, if the “image” of high sensitivity is dominated by the perception of nuances, subtleties, and positive emotions related to art, i.e., an individual is characterized by a rich inner life, self-awareness, and empathy toward others, these may influence adaptive potential and resistance in difficult situations. Additionally, the significant positive correlation between *Sensing the Subtle* and *Mindful Attention Awareness* may indicate that people with a high score in this HSP subscale are more focused on “here and now” and conscious presence; also, they are more aware of stimuli from the external and internal environment. Developing mindfulness can significantly contribute to counterbalancing negative emotionality and can influence individual wellbeing.

### Regression Analysis

Regression analysis revealed that SPS and attention awareness are important predictors of selected aspects of resilience (Table 2). Consequently, the *Emotional Reactivity* and *Sensing the Subtle* HSPS subscales are significant predictors of general resilience and each tested aspect of resilience. They also influence resilience in an opposite way: Emotional reactivity has a negative influence, while ability to sense the subtle (StS) has a positive impact. Additionally, the *Overstimulation* subscale was found to be a significant predictor of general resilience and its subscales: *Determination and persistence in actions* and *Optimistic life attitude and ability to mobilize in difficult situations*. Interestingly, in both cases, it had a positive influence on resilience. Mindful attention awareness was shown to be a significant predictor of the *Openness to new experiences and a sense of humor* and *Tolerance of failures and treating life as a challenge* resilience subscales, on which it had a positive impact. Thus, the results of the regression analysis confirm hypothesis 4 (H4: *Sensory processing sensitivity and mindful attentional awareness are significant predictors of resilience*).

**Table 2:** Multiple regression analysis for variables predicting general resilience and its subscales (*N*=273).

		*B*	Std. Error	Beta	*t*	*p*	95% confidence interval of the B	
Model for Resilience	**Variables**						Lower	Upper
	Constant	2.891	0.277		10.441	<0.001	2.346	3.436
	ER	−0.421	0.044	−0.613	−9.470	<0.001	−0.508	−0.333
	StS	0.241	0.039	0.346	6.216	<0.001	0.165	0.318
	OvSt	0.070	0.033	0.142	2.153	0.032	0.006	0.134
	MAAS	0.041	0.041	0.052	1.005	0.316	−0.040	0.123
*R*=0.574, *R*^2^=0.330, Adj. *R*^2^=0.320								
**Model for Determination and persistence in actions**	
								
	Constant	1.363	0.399		3.412	0.001	0.577	2.149
	ER	−0.382	0.064	−0.407	−5.954	<0.001	−0.508	−0.255
	StS	0.384	0.056	0.403	6.858	<0.001	0.274	0.495
	OvSt	0.147	0.047	0.218	3.132	0.002	0.055	0.239
	MAAS	0.066	0.060	0.061	1.116	0.266	−0.051	0.184
*R*=0.502, *R*^2^=0.252, Adj. *R*^2^=0.241								
**Model for Openness to new experiences and a sense of humor**	
								
	Constant	3.112	0.322		9.671	<0.001	2.478	3.745
	ER	−0.301	0.052	−0.413	−5.837	<0.001	−0.403	−0.200
	StS	0.187	0.045	0.251	4.135	<0.001	0.098	0.275
	OvSt	0.006	0.038	0.012	0.170	0.865	−0.068	0.081
	MAAS	0.109	0.048	0.129	2.273	0.024	0.015	0.203
*R*=0.449, *R*^2^=0.202, Adj. *R*^2^=0.190								
**Model for Competencies to cope and tolerance of negative affect**	
								
	Constant	3.704	0.377		9.831	<0.001	2.962	4.446
	ER	−0.512	0.060	−0.579	−8.469	<0.001	−0.631	−0.393
	StS	0.235	0.053	0.261	4.448	<0.001	0.131	0.339
	OvSt	0.071	0.044	0.112	1.613	0.108	−0.016	0.159
	MAAS	−0.076	0.056	−0.074	−1.346	0.179	−0.186	0.035
*R*=0.503, *R*^2^=0.253, Adj. *R*^2^=0.242								
**Model for Tolerance of failures and treating life as a challenge**	
	Variables						Lower	Upper
	Constant	2.605	0.344		7.564	<0.001	1.927	3.283
	ER	−0.358	0.055	−0.444	−6.470	<0.001	−0.466	−0.249
	StS	0.213	0.048	0.260	4.417	<0.001	0.118	0.308
	OvSt	0.017	0.040	0.029	0.409	0.683	−0.063	0.096
	MAAS	0.169	0.051	0.181	3.301	0.001	0.068	0.270
*R*=0.498, *R*^2^=0.248, Adj. *R*^2^=0.237								
**Model for Optimistic life attitude and ability to mobilize in difficult situations**	
								
	Constant	3.670	0.396		9.276	<0.001	2.891	4.449
	ER	−0.551	0.063	−0.597	−8.680	<0.001	−0.676	−0.426
	StS	0.188	0.055	0.200	3.382	0.001	0.078	0.297
	OvSt	0.109	0.046	0.164	2.338	0.020	0.017	0.200
	MAAS	−0.062	0.059	−0.058	−1.050	0.294	−0.178	0.054
*R*=0.495, *R*^2^=0.245, Adj. *R*^2^=0.234								

*ER – Emotional Reactivity; StS – Sensing the Subtle; OvSt – Overstimulation; and MAAS – Mindful Attention Awareness (N=273)*.

### Moderation Analysis

**Figure 3:**
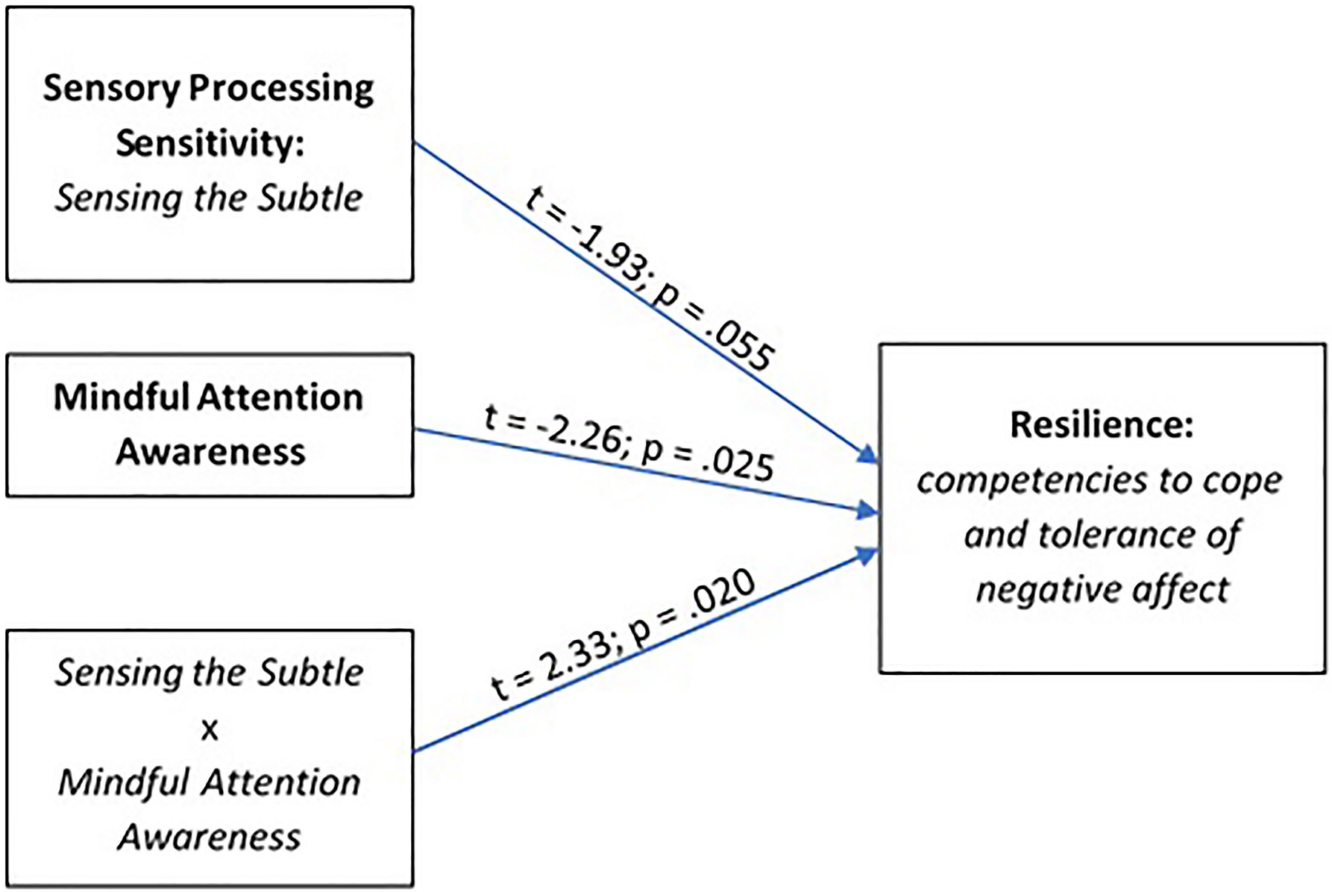
The model of relations between SPS (*Sensing the Subtle* subscale) and resilience (*Competencies to cope and tolerance of negative affect* subscale) moderated by mindful attention awareness.

In the next step, moderation analysis was performed to evaluate the role of mindfulness in explaining how individual cognitive dispositions influence the relationship between temperamental characteristics associated with SPS and resilience. The type of model 1 (Hayes, 2018) was tested in numerous configurations in which sensitiveness (HSPS, ER, StS, and OvSt) was an exposure variable, mindful attention awareness was a moderator, and resilience and its different aspects (DPA, OH, CNA, TFC, and OM) were an outcome variable. Figure 3 presents a model in which a significant interaction effect was observed as: Mindful attention awareness was a significant variable that strengthens the influence of the ability to StS on the coping with negative affect (CNA) competence, *F* (3,269)=2.927, *p* =0.034, *R*^2^=0.032. The moderation analysis revealed that the moderator value that defines the Johnson-Neyman significance region (Hayes, 2018) was 3.979. When this value is higher, the higher the mindful attention awareness score, the stronger the positive interaction effect of sensing the subtle and attention awareness on coping and tolerance of negative affect.

This analysis shows that attentional awareness is a significant moderator between a selected aspect of SPS, namely, sensing the subtle, and a selected aspects of resilience, namely, competencies to cope and tolerate negative affect; this partially supports hypothesis 5 (H5: *Attentional awareness is an important factor that moderates the relationship between sensory processing sensitivity and resilience*).

## Discussion

The presented study focused on analyzing the relationship between sensitivity and resilience, taking into account cognitive aspects that may be important moderator of these relationships. We analyzed these relationships in a group of young adults during their transition to the labor market. The results showed significant relationships between SPS, attention awareness, and resilience. Specifically, the results indicate negative associations between emotional reactivity and resilience; and a positive association between sensing the subtle, mindful attention awareness, and resilience. The results of correlation analysis showed that some aspects of high SPS are individual resources that may significantly decrease resilience (i.e., emotional reactivity), while other (i.e., sensing the subtle) may increase it or have an ambiguous effect (i.e., overstimulation). Other research also presents some ambiguous associations between specific aspects of sensitivity and resilience. For example, research on high interpersonal sensitivity and resilience in young adults showed that the key moderating factor is the need for social approval, which can be a risk factor when it is high or a protective factor when it is low (Aydogdu et al., 2017). These findings indicate that interpersonal sensitivity can have two effects on resilience: It can increase or decrease mental toughness, depending on other individual characteristics.

Regression analysis showed that SPS is an important predictor of general resilience and each tested aspect of resilience: Emotional reactivity has a negative influence, while ability to StS has a positive effect. The tendency to be overstimulated was found to be a significant predictor of general resilience and its two subscales: *Determination and persistence in actions* and *Optimistic life attitude and ability to mobilize in difficult situations*. In both cases, it had a positive influence on resilience that could be linked with awareness of one’s state and the ability to develop functional regulatory strategies in a state of overstimulation. As expected, mindful attention awareness was a significant predictor of resilience but only in two its aspects: *Openness to new experiences and a sense of humor* and *Tolerance of failures and treating life as a challenge*. This may lead to the conclusion that cognitive dispositions and strategies are of high importance in coping with stress and for improving individual wellbeing.

The results of the moderation analysis indicate that attention awareness training may be particularly helpful for highly sensitive persons as conscious presence significantly moderates the relation between sensing the subtle and CNA. Mindfulness training may be a substantial regulation strategy that can help cope with overstimulation and the consequences of high emotional reactivity. The importance of mindfulness training was demonstrated by Amemiya et al. (2020). A group of graduate students with high and low SPS participated in a yoga course. Mood and level of attention control were analyzed. Prior to starting the yoga course, the highly sensitive participants had lower attention control scores and a more negative mood than the less sensitive participants. However, attention control and mood scores improved in the group of highly sensitive participants after the yoga course. Although high SPS is associated with mental health risks, including symptoms of anxiety and depression (Liss et al., 2005), effective methods of regulation can be obtained by developing cognitive competences, especially communication skills, decision-making skills, and emotional coping skills (Yano et al., 2021). Yoga promotes attention and emotional control, therefore it can effectively counteract the feeling of being overwhelmed or overstimulated, consequently improving mood. The beneficial importance of yoga for mental health is also indicated by Büssing et al. (2012).

Complex effects related to high sensitivity were discussed in research that indicated the significant influence of external conditions. Boyce and Ellis (2005) indicate that reactivity to stress is not a linear process that leads to increased arousal; it also includes circuits that are used to modify or alleviate it. Depending on the context, hyperresponsive phenotypes may have positive and negative consequences, i.e., both risk-increasing and protective effects. The effects of highly responsive phenotypes are more bivalent than univalent; they have both risk-increasing and protective effects, depending on the context. According to Boyce and Ellis (2005), increased stress responsiveness reflects biological context sensitivity; it has potentially negative effects under adversity and positive effects under supportive and protective conditions. This is in line with the findings of De Villers et al. (2018), who indicate that highly sensitive persons may benefit much more from positive interventions than less sensitive people; this includes responses to psychotherapeutic intervention and the influence of social support.

High sensitivity and its possible consequences for an individual may be analyzed in context of stress models. Belsky and Pluess (2009) note that the *Diathesis-Stress* model (Monroe and Simons, 1991) focuses on individual differences (risk factors) in exposure and susceptibility to stressful situations, while the concept of *Differential Susceptibility* (Belsky et al., 2007) includes both negative and positive responses to environmental requirements. In the *Diathesis-Stress* concept, non-susceptibility equates to resistance. Within the framework of the *Differential Susceptibility* theory, Pluess and Belsky (2013) propose the *Vantage Sensitivity* model, which focuses on the individual differences (protective factors) that give an adaptive advantage as a result of benefiting from positive experiences. The *Vantage Sensitivity* model focuses on the benefits of positive interactions, while resilience stands for *Vantage Resistance*, which focuses on the benefits of coping with negative experiences. In these terms, highly sensitive sensory processing offers an adaptive advantage not only in terms of the “control pause” (stopping an action, analyzing situations, and reflecting), but also by making full use of positive experiences. The reference to the *Vantage Sensitivity* concept makes it possible to treat high SPS as a resource that allows one to take full advantage of positive exposures, focus on the positive aspects of events, and refer to positive past experiences in new situations.

Wyller et al. (2017) proposed a model whose basic thesis is the assumption that the negative effects of stress are not caused by the sensitivity of sensory processing itself: Instead, maladaptive content and overwhelming emotions are considered to be the result of cognitive reactivity. In the concept of cognitive reactivity, attention shifts from external stressors to internal negative thoughts, beliefs, or prejudices. This indicates that distress can occur even without exposure to strong negative environmental stimuli. Highly sensitive people perceive negative stimuli more intensely, which in turn intensifies negative emotions and negative cognitive processing, thus creating vicious circles that result in symptoms of anxiety and depression or somatic complaints. Emotional regulation strategies mediate between the sensitivity of sensory processing and psychological distress. Psychological interventions that target cognitive reactivity have a greater chance of success than interventions aimed at SPS, which is difficult to modify. Wyller et al. (2017) propose increasing the ability to shorten “self-propelling erroneous cycles” as the main goal of the intervention.

Harms et al. (2019) oppose perceiving high sensitivity in terms of weakness and indicate that highly sensitive people can be very entrepreneurial because they process environmental and social stimuli more deeply and perceive social signals faster: They can quickly recognize opportunities; they are empathetic and creative. By engaging in entrepreneurship, they can shape their work environment in a way that suits them, e.g., by determining the rhythm of work and the level of workload. As a result, they may be more independent and successful. The research of Harms et al. (2019) showed relationships of complex causality. The ability to recognize opportunity turned out to be a core factor, and its combination with SPS or with the entrepreneurial trait profile created sufficient conditions for the emergence of entrepreneurial intention. Traditionally, entrepreneurs are viewed as extroverted, open-minded, and conscientious, but the meta-trait of high sensitivity creates an alternative path. The necessity to adjust the working environment to the unique needs of highly sensitive people may stimulate their willingness to act as entrepreneurs. SPS reflects perceived desirability, while the ability to recognize opportunities reflects perceived feasibility. Both these aspects reflect the entrepreneurial trait profile and are crucial in achieving goals.

The sample consisted of young adults, mostly at the stage of entering the labor market. In this period, highly sensitive young people who are leaving education and looking for employment may experience a high level of stress due to, for example, social exposure, assessments, and potential rejection by prospective employers. Since high SPS can result in a high level of distress, anxiety, and a sense of overload, the search for protective factors is important in order to maintain the wellbeing and efficient functioning of highly sensitive people, especially in relation to potentially stressful situations, such as transition to the labor market. This process can be viewed in terms of self-regulatory, autonomous, goal-oriented, and proactive processes (Van Hooft et al., 2013; Wanberg et al., 2020). The three dimensions of a job search are the effort put into it, the quality of the search (activities in which the person engages), and persistence, which means the continuity or variability of efforts over time. Jobseekers must develop a strategy for daily action plans; they must also motivate themselves and initiate or modify their behavior based on feedback from the environment. Contextual factors (such as the unemployment rate in the region, the specificity of existing workplaces, the level of economic development, and cultural conditions), factors relating to the professional situation, and the preferences of employers should be taken into consideration. Additionally, in order to achieve success, jobseekers should control negative emotions, maintain openness to new suggestions, and strengthen their sense of internal control and self-efficacy, all of which are helpful in maintaining a persistent pursuit of the goal. According to Van Hooft et al. (2013), the four-stage cyclical self-regulatory model of the job search process includes the following phases:

- goal establishment (selecting a goal, goal commitment, goal clarity, and organized goal hierarchy).
- planning goal pursuit (strategy selection, selecting and forming intentions, prioritizing, preparation, deadline setting, and forming implementation intentions).
- goal striving (self-control of attention, thoughts, emotions, motivation, behavior, goal maintenance, self-monitoring, and active feedback seeking).
- reflection (evaluation of performance in the light of the established goals, attribution of potential failures to changeable causes, learning from failures, and self-rewarding).

The variables included in this model are largely related to the abilities to reflect, focus attention, self-motivate, emotionally regulate, and learn from experience. On the one hand, highly sensitive people, due to their excessive emotional reactivity and tendency to be overstimulated, may experience failures more strongly, become discouraged easily, or be overwhelmed by the novelty of the situation and excessive stimulation. However, regarding their deep processing, they may also be fully aware of their emotions, regulate their intensity and duration, distance themselves from the situation, present it in a wider perspective, relate current goals to priorities, refer to their values in goal selection, or change the situational context. The ability to perceive demanding or difficult situations in a nuanced way may constitute an adaptive resource. It is possible that perception of difficult situations depends on both early experiences (as suggested by Aron, 2017) and on competences related to mindfulness, thus facilitating the reception of external and internal stimuli and their deep and reflective multi-level analysis. This may develop the potential of a highly sensitive persons, thus improving their wellbeing and health.

According to research, highly sensitive persons may constitute about 25–30% of the population (Aron and Aron, 1997; Lionetti et al., 2018) and may cope worse than others in difficult situations if their sensitivity is not accompanied by mental resilience. Elst et al.’s (2019) analysis of the professional situations of highly sensitive people showed that high sensitivity should be treated as both a personal resource and as a risk factor that increases susceptibility to work-related stress. High sensitivity can strengthen the relationship between job demands and the feeling of exhaustion; it can do the same for the relationship between work resources and supportive behavior toward co-workers. In Elst et al.’s research, EOE and LST were found to positively moderate the relationship between job demands and emotional exhaustion. However, low sensory threshold positively moderated the relationship between resources in the workplace and supportive behaviors. The role of each of the three components of high sensitivity turned out to be different. Bakker et al. (2014) analyzed high SPS in the context of the Job Demands-Resources model. Elst et al. (2019) emphasized that the high-sensitivity trait may act as both a risk factor and a personal resource, depending on the perceived nature of the work environment. Employees with a high level of SPS react more strongly not only to negative aspects of the work environment but also to positive experiences, relationships, and circumstances, which can they therefore use more fully than others. Under optimal conditions, highly sensitive individuals can function excellently, but when faced with increased professional demands they may feel overwhelmed and overstimulated. Highly sensitive people are therefore more susceptible to the conditions prevailing in the work environment. Elst et al.’s study provided evidence for the phenomenon of differential susceptibility to the work environment and developed the Job Demands-Resources model by adding SPS as a new feature that is essential for employee functioning in the workplace.

The study has raised a number of methodological questions. High sensitivity, tested by Aron and Aron’s (1997) scale, includes two subscales related to emotionality (12 *Emotional Reactivity* items and 7 *Overstimulation* items) and only one scale referring to the perception of nuances and subtleties and deeper processing of stimuli (6 *Sensing the Subtle* items). The proportion of items indicates that the overall HSPS score is influenced by two scales reflecting aspects of emotional sensitivity (*Emotional Reactivity* and *Overstimulation*), which may be close to neuroticism in the case of high scores (Evans and Rothbart, 2008, 2009). On the other hand, the potential advantage of high sensitivity may be underestimated as there is only one *Sensing the Subtle* subscale, which has the least items. Moreover, the positive and negative effects of high sensitivity may neutralize each other; therefore, it is particularly important to specify different aspects of SPS instead of analyzing the overall HSPS score. The other aspects that are important to emphasize are the negative way in which questions are formulated (Greven et al., 2019) and the inconsistent results of factor analyses in various studies (e.g., Aron and Aron, 1997; Smolewska et al., 2006; Evans and Rothbart, 2008; Blach, 2015; Lionetti et al., 2018). It seems valuable to specify the nature, strength, or quality of a stimulus that is considered to be discomforting for the examined person. In addition, it seems that the measure of high sensitivity would be stronger if it could examine the “control pause” mechanism. For further studies, it would be valuable to explore physiological indicators of SPS in different tasks and contexts. Moreover, as noted by Wyller et al. (2017), neither the heterogeneity within the high-sensitivity category nor the variability of individual sensitivity over time are taken into account in theory and research; the development of interventions that could facilitate the functioning of highly sensitive people would be more practical if a certain level of plasticity in terms of the phenotype was assumed.

### Limitations

The limitations of the presented research are associated with the characteristics of the study sample, which consists of young adults (mainly students) and is not representative. The obtained results should be verified in a sample that is more varied in terms of gender, age, and employee status. The study sample consists mainly of women; therefore, in future studies, it is important to balance the ratio of women and men. The limitations are related to the online character of the study and the lack of control over completion of the survey. Additionally, we used self-report questionnaires, which may be biased by current emotional state and expectations. In future research, it would be valuable to test the moderating effect of attention awareness in the experimental design by analyzing how highly sensitive persons may be supported through cognitive training in, e.g., demanding contexts or stressful situations, and analyze how such cognitive training may influence their stress response.

### Conclusion

The results indicate significant relationships between SPS and resilience, and a significant moderating role of mindful attention awareness. Mindfulness was found to strengthen the association between the ability to StS and to tolerate negative affect. Thus, it may be particularly important to involve highly sensitive employees in training dedicated to attention awareness and conscious presence. Highly sensitive persons with the dominant characteristics of high emotional reactivity and a tendency to be overstimulated may especially need support to take advantage of the protective factors associated with high sensitivity, i.e., sensing the subtle, detecting nuances, and seeing things from different perspectives, all of which result in deeper analysis and greater reflectivity. Applying cognitive strategies in stress-related contexts may be crucial for coping with difficulties and improving individual wellbeing.

## Data Availability Statement

The raw data supporting the conclusions of this article will be made available by the authors, without undue reservation.

## Ethics Statement

The studies involving human participants were reviewed and approved by the Research Ethics Committee of the Institute of Applied Psychology, Jagiellonian University in Kraków. Written informed consent for participation was not required for this study in accordance with the national legislation and the institutional requirements.

## Author Contributions

BG and KG: substantial contributions to the conception and design of the work, acquisition, analysis, interpretation of data, drafting the work, revising it critically, final approval of the version to be published, and agrees to be accountable for all aspects of the work. All authors contributed to the article and approved the submitted version.

## Funding

The open access license of the publication was funded by the Priority Research Area Society of the Future under the programme “Excellence Initiative–Research University” at the Jagiellonian University in Kraków.

## Conflict of Interest

The authors declare that the research was conducted in the absence of any commercial or financial relationships that could be construed as a potential conflict of interest.

## Publisher’s Note

All claims expressed in this article are solely those of the authors and do not necessarily represent those of their affiliated organizations, or those of the publisher, the editors and the reviewers. Any product that may be evaluated in this article, or claim that may be made by its manufacturer, is not guaranteed or endorsed by the publisher.
